# Supplementary material for: Translation of Mutant Repetitive Genomic Sequences in Hirsutella sinensis and Changes in the Secondary Structures and Functional Specifications of the Encoded Proteins
Source: Int J Mol Sci. 2024 Oct 17;25(20):11178. doi: 10.3390/ijms252011178 (PMC11508423; doi:10.3390/ijms252011178)
Supplement: Supplementary file 1 [file ijms-25-11178-s001.zip › ijms-3236691-supplementary.pdf]

Translation of Mutant Repetitive Genomic Sequences in *Hirsutella sinensis* and Changes in the Secondary Structures and Functional Specifications of the Encoded Proteins

Xiu-Zhang Li [1], Yu-Ling Li [1], Ya-Nan Wang [2] and Jia-Shi Zhu [1,3,*]

[1] State Key Laboratory of Plateau Ecology and Agriculture, Qinghai Academy of Animal Science and Veterinary, Qinghai University, Xining 810016, China; xiuzhang11@163.com or xiuzhang@qhu.edu.cn (X.-Z.L.); yulingli2000@163.com or 1991990033@qhu.edu.cn (Y.-L.L.)
[2] State Key Laboratory of Quality Ensurance and Sustainable Use of Dao-di Herbs, National Resource Center for Chinese Materia Medica, China Academy of Chinese Medical Sciences, Beijing 100700, China; wangyn@nrc.ac.cn
[3] Institute of Biopharmaceutical and Health Engineering, Shenzhen International Graduate School, Tsinghua University, Shenzhen 518055, China
* Correspondence: zhujosh@163.com

Table S1. Amino acid scales for ProtScale plotting to predict secondary structures (α-helices, β-turns, and coils [55] and β-sheets [54]) of proteins.

			Chemical-physical property	α-Helix	β-Sheet	β-Turn	Coil
Aspartic acid	Asp	D	Acidic	0.924	0.540	1.197	1.197
Glutamic acid	Glu	E	Acidic	1.504	0.370	1.149	0.761
Alanine	Ala	A	Aliphatic	1.489	0.830	0.788	0.824
Isoleucine	Ile	I	Aliphatic	1.003	1.600	0.240	0.886
Leucine	Leu	L	Aliphatic	1.236	1.300	0.670	0.810
Valine	Val	V	Aliphatic	0.990	1.700	0.387	0.772
Phenylalanine	Phe	F	Aromatic	1.195	1.380	0.624	0.797
Tryptophan	Trp	W	Aromatic	1.090	1.370	0.546	0.941
Tyrosine	Tyr	Y	Aromatic	0.787	1.470	0.795	1.109
Arginine	Arg	R	Basic	1.224	0.930	0.912	0.893
Histidine	His	H	Basic	1.003	0.870	0.970	1.068
Lysine	Lys	K	Basic	1.172	0.740	1.302	0.897
Asparagine	Asn	N	with polar neutral side chains	0.772	0.890	1.572	1.167
Cysteine	Cys	C	with polar neutral side chains	0.966	1.190	0.965	0.953
Glutamine	Gln	Q	with polar neutral side chains	1.164	1.100	0.997	0.947
Methionine	Met	M	with polar neutral side chains	1.363	1.050	0.436	0.810
Serine	Ser	S	with polar neutral side chains	0.739	0.750	1.316	1.130
Threonine	Thr	T	with polar neutral side chains	0.785	1.190	0.739	1.148
Glycine	Gly	G	Unique amino acids	0.510	0.750	1.860	1.251
Proline	Pro	P	Unique amino acids	0.492	0.550	1.415	1.540

Table S2: Authentic *H. sinensis* genes for the heavy metal tolerance protein precursor (the query sequence) and the repetitive genomic copies with major decrease (> 5%) in the AT content.

Table S2: Authentic *H. sinensis* genes for the heavy metal tolerance protein precursor (the query sequence) and the repetitive genomic copies with major decrease (>5%) in the AT content.

The subject sequence (repetitive copy)		*vs*. The query sequence (195005→198178 of NGJJ01001482 of the authentic gene for the heavy metal tolerance protein precursor)		Mutation in the subject sequence compared with the query sequence						Transcript in the mRNA transcriptome GCQL00000000			
				Transitions		Transversions		Total point mutations					
H. sinensis strain	Genomic fragment (sequence range)	Similarity	Change in AT content	C-to-T and G-to-A	T-to-C and A-to-G	C-to-A and G-to-T	A-to-C and T-to-G	G or C to A or T	A or T to G or C	Transcriptomic fragment	Similarity	Query coverage	Translated protein sequence
1229	LKHE01001285 (2772←5944)	99.97%	Nearly no Δ (41.2%)							GCQL01012210 (442←1471)<br>GCQL01006458 (2←683)<br>GCQL01000615 (1←403)	100%<br>99.9%<br>100%	32%<br>21%<br>12%	EQK98634 (1→344)<br>EQK98634 (572→798)<br>EQK98634 (796→929)
	LKHE01002445 (3819←4233)	65.4%	↓ 39.5% to 33.3%	14	26	14	27	28	53	GCQL01017466 (726←1140)	100%	100%	EQK98286 (378→515)
	LKHE01001747 (128317←128839; 128885←129014)	67.4%	↓ 38.3% to 34.6%	24	34	21	34	45	68	GCQL01013313 (1←426)<br>GCQL01016959 (1→271)	99.8%<br>100%	61% 38%	EQK99315 (592→732)<br>EQK99315 (734→823)
CC1406-203	NGJJ01001482 (195005→198178)	100%	No Δ (40.2%)							GCQL01012210 (442←1471)<br>GCQL01006458 (2←683)<br>GCQL01000615 (1←403)	100%<br>99.9%<br>100%	32% 21%<br>12%	EQK98634 (1→344)<br>EQK98634 (572→798)<br>EQK98634 (796→929)
	NGJJ01001243 (150283→150697)	65.4%	↓ 39.5% to 33.3%	14	26	14	27	28	53	GCQL01017466 (726←1140)	100%	100%	EQK98286 (378→515)
	NGJJ01001310 (461774←461903; 461949←462471)	67.4%	↓ 38.3% to 34.6%)	24	34	21	34	45	68	GCQL01013313 (1→426)<br>GCQL01016959 (1←271)	99.8%<br>100%	61% 38%	EQK99315 (592→732)<br>EQK99315 (734→823)
Co18	ANOV01008830 (2485←5657)	99.97%	Nearly no Δ (40.2%)							GCQL01012210 (442←1471)<br>GCQL01006458 (2←683)<br>GCQL01000615 (1←403)	100%<br>99.9%<br>100%	32% 21%<br>12%	EQK98634 (1→344)<br>EQK98634 (572→798)<br>EQK98634 (796→929)
	ANOV01010958 (3175→3589)	65.4%	↓ 39.5% to 33.3%	14	26	14	27	28	53	GCQL01017466 (726←1140)	100%	100%	EQK98286 (378→515)
	ANOV01005573 (4224←4746; 4792←4921)	67.4%	↓ 38.3% to 34.6%	24	34	21	34	45	68	GCQL01013313 (1←426)<br>GCQL01016959 (1→271)	99.8%<br>100%	61% 38%	EQK99315 (592→732)<br>EQK99315 (734→823)
IOZ07	JAAVMX010000003 (1309808←1312980)	99.97%	Nearly no Δ (40.2%)							GCQL01012210 (442←1471)<br>GCQL01006458 (2←683)<br>GCQL01000615 (1←403)	100%<br>99.9%<br>100%	32%<br>21%<br>12%	EQK98634 (1→344)<br>EQK98634 (572→798)<br>EQK98634 (796→929)
	JAAVMX010000002 (833901←834315)	65.4%	↓ 39.5% to 33.3%	14	26	14	27	28	53	GCQL01017466 (726←1140)	100%	100%	EQK98286 (378→515)
	JAAVMX010000012 (85112←85634; 85680←85809)	67.4%	↓ 38.3% to 34.6%	24	34	21	34	45	68	GCQL01013313 (1←426)<br>GCQL01016959 (1→271)	99.8%<br>100%	61%<br>38%	EQK99315 (592→732)<br>EQK99315 (734→823)
ZJB12195	LWBQ01000001 (1338987←1342159)	99.97%	Nearly no Δ (41.2%)							GCQL01012210 (442←1471)<br>GCQL01006458 (2←683)<br>GCQL01000615 (1←403)	100%<br>99.9%<br>100%	32%<br>21%<br>12%	EQK98634 (1→344)<br>EQK98634 (572→798)<br>EQK98634 (796→929)
	LWBQ01000010 (468456←468870)	65.4%	↓ 39.5% to 33.3%	14	26	14	27	28	53	GCQL01017466 (726←1140)	100%	100%	EQK98286 (378→515)
	LWBQ01000044 (141513→142035; 142081→142210)	67.4%	↓ 38.3% to 34.6%	24	34	21	34	45	68	GCQL01013313 (1←426)<br>GCQL01016959 (1→271)	99.8%<br>100%	61%<br>38%	EQK99315 (592→732)<br>EQK99315 (734→823)

Note: "→" and "←" indicate the sequence directions of the sense and antisense chains, respectively. "↓" indicates a decrease in the AT content.

Table S3: Authentic *H. sinensis* genes for the maltose permease (query sequence) and repetitive genomic copies with minor increases (≤ 5%) in the AT content.

Table S3: Authentic *H. sinensis* genes for the maltose permease (query sequence) and repetitive genomic copies with minor increases (≤5%) in the AT content.

The subject sequence (repetitive copy)		*vs.* The query sequence (47693→49623 of LKHE01002657 of the authentic gene for the maltose permease)		Mutation in the subject sequence compared with the query sequence						Transcript in the mRNA transcriptome GCQL00000000			
				Transitions		Transversions		Total point mutations					
H. sinensis strain	Genomic fragment (sequence range)	Similarity	Change in AT content	C-to-T and G-to-A	T-to-C and A-to-G	C-to-A and G-to-T	A-to-C and T-to-G	G or C to A or T	A or T to G or C	Transcriptomic fragment	Similarity	Query coverage	Translated protein sequence
1229	LKHE01002657 (47693→49623)	100%	No Δ (40.1%)							GCQL01010488 (586→1622)	99.9%	80%	EQK98556 (189→533)
	LKHE01000336 (6824→7385)	69.6%	↑ 37.9% to 39.3%	30	35	28	11	58	46	GCQL01015773 (948→1509) or GCQL01019033 (526→1063) with a 25 nt deletion	100%<br>95.6%	100%<br>100%	EQL02995 (152→338)<br>EQL02995 (152→338)
CC1406-203	NGJJ01000203 (77988→79917)	99.8%	Nearly no Δ (↑ 40.1% to 40.2%)	1	0	0	0	1	0	GCQL01010488 (334→1892)	99.9%	80%	EQK98556 (189→533)
	NGJJ01001217 (782871←782913; 1076399←1076960)	70.4%	↑ 36.8% to 38.5%	31	37	28	10	59	47	GCQL01015773 (948→1509) or GCQL01019033 (526→1063) with a 25 nt deletion	100%<br>95.6%	100%<br>100%	EQL02995 (152→338)<br>EQL02995 (152→338)
Co18	ANOV01009266 (1725←3655)	99.8%	Nearly no Δ (↓ 40.1% to 40.0%)	1	2	0	0	1	2	GCQL01010488 (586→1622)	99.8%	80%	EQK98556 (189→533)
	ANOV01000509 (4433←4994, 5254←5290)	70.1%	↑ 37.9% to 39.9%	31	37	28	10	59	47	GCQL01015773 (948→1509) or GCQL01019033 (526→1063) with a 25 nt deletion	100%<br>95.6%	100%<br>100%	EQL02995 (152→338)<br>EQL02995 (152→338)
IOZ07	JAAVMX010000006 (4614206→4616136)	99.9%	Nearly no Δ (40.1%)	1	0	0	0	1	0	GCQL01010488 (334←1892)	100%	80%	EQK98556 (189→533)
	JAAVMX010000005 (12448464→12449025, 12779815→12779864)	70.4%	↑ 37.6% to 39.2%	31	37	28	10	59	47	GCQL01015773 (948→1509) or GCQL01019033 (526→1063) with a 25 nt deletion	100%<br>95.6%	100%<br>100%	EQL02995 (152→338)<br>EQL02995 (152→338)
ZJB12195	LWBQ01000157 (66747→68677)	99.9%	Nearly no Δ (40.1%)	1	0	0	0	1	0	GCQL01010488 (586→1622)	100%	80%	EQK98556 (189→533)
	LWBQ01000003 (292175→292217, 570642→571203)	70.4%	↑ 36.8% to 38.5%	30	35	28	11	58	46	GCQL01015773 (948→1509) or GCQL01019033 (526→1063) with a 25 nt deletion	100%<br>95.6%	100%<br>100%	EQL02995 (152→338)<br>EQL02995 (152→338)

Note: "→" and "←" indicate the sequence directions of the sense and antisense chains, respectively. "↑" and "↓" indicate increases and decreases in the AT content, respectively.

Table S4. Percent similarities between nrDNA ITS1-5.8S-ITS2 sequences of 5 genome assemblies of Genotype #1 *H. sinensis* and 17 *O. sinensis* genotype sequences. (Modified from Table 1 of [5]).

Genotype # and GenBank accession #	Percent similarity between the *H. sinensis* genomic ITS segments and 17 *O. sinensis* genotypes				
	ANOV01021709 838→1504	LKHE01000582 2074→2740	LWBQ01000008 991739→992405	JAAVMX010000017 9029←9695	NGJJ01000799 3237→3903
#1 AB067721	99.7%	100%	99.6%	100%	100%
#2 MG770309	94.4%	94.7%	94.7%	94.7%	94.7%
#3 HM595984	95.9%	96.1%	95.6%	95.3%	95.3%
#7 AJ488254	94.7%	95.0%	95.3%	95.0%	95.0%
#8 GU246286	89.5%	89.5%	89.2%	89.5%	89.5%
#9 GU246288	95.0%	95.2%	94.6%	95.2%	95.2%
#10 GU246287	83.0%	83.2%	83.0%	83.2%	83.2%
#11 JQ695935	78.9%	79.1%	78.5%	79.1%	79.1%
#12 GU246296	94.3%	94.5%	93.9%	94.5%	94.5%
#13 KT339190	87.5%	87.5%	87.2%	87.5%	87.5%
#14 KT339178	89.3%	89.6%	89.1%	89.6%	89.6%
#4 AB067744	89.1%	89.4%	89.3%	89.4%	89.4%
#5 AB067740	86.3%	86.7%	86.2%	86.7%	86.7%
#6 KJ720572	85.5%	85.5%	85.5%	85.5%	85.5%
#15 KT232017	89.6%	89.9%	89.4%	89.9%	89.9%
#16 KT232019	87.4%	87.7%	87.6%	87.7%	87.7%
#17 KT232010	87.7%	88.1%	87.5%	88.1%	88.1%

Note: "GT" represents the genotype. GC-biased genotypes are listed in blue on the upper panel. AT-biased genotypes are listed in red on the lower panel. The GenBank accession numbers are hyperlinked to the GenBank database.

```
LWBQ01000158  32562   CCTCCCCCATCATGGCGGGGCGAAGCACCCCCTCGGGACCCGGACACCCGTCTGATTCGC  32621
LWBQ01000021  415851  ------------------------------------------------------------  415792
GCQL01000547  610     ------------------------------------------------------------  669

LWBQ01000158  32622   AGTACGGCGAGGAATGGGCCCAGGACCTGCGCATCAAGTTCGAGGGCCTGCTGCGCGACA  32681
LWBQ01000021  415791  ------------------------------------------------------------  415732
GCQL01000547  670     ------------------------------------------------------------  729

LWBQ01000158  32682   AGCGCATGACCGACTTGCGCACCACCTCGAGCCATGCTTCTCCCGGCGTCACGGAGCGGT  32741
LWBQ01000021  415731  ------------------------------------------------------------  415672
GCQL01000547  730     ------------------------------------------------------------  789

LWBQ01000158  32742   CATCGAGCGCAAACCTCCGCGCCCGTCCCGCCGCCAACGACGCTC                 32786
LWBQ01000021  415671  ---------------------------------------------GGCCTGCCACCGCCC  415612
GCQL01000547  790     ---------------------------------------------                 834

LWBQ01000158  32787                                         GGCCTGCCACCGCCCACGGTCA  32808
LWBQ01000021  415611  ACGGTCAAGCCACTCCTCCGCCTCCATTCGTGCATGCC----------------------  415552
GCQL01000547  835                                           ----------------------  856

LWBQ01000158  32809   AGCCACTCCTCCGCCTCCATCCTACTCGTGCATGCGCCATCTTCCCATGATCCCGACTGC  32868
LWBQ01000021  415551  ------------------------------------------------------------  415492
GCQL01000547  857     ------------------------------------------------------------  916

LWBQ01000158  32869   GCCAGACGCCGCCGACCGCGACTCCCAAAAGTTCCGCAACCTCCTCATCAGCCTGTCGTT  32928
LWBQ01000021  415491  ------------------------------------------------------------  415432
GCQL01000547  917     ------------------------------------------------------------  976

LWBQ01000158  32929   GACGCCCACAAAGTACGAAAACCCCGGCCTCCTCGACGAGGCCCTCTTGAAAATTCCTCT  32988
LWBQ01000021  415431  ------------------------------------------------------------  415372
GCQL01000547  977     ------------------------------------------------------------  1036

LWBQ01000158  32989   CGATCGCATCTACGCTGAGGCCGAGGAGGAGGTTCAGGTGCTCCAGGCCGAGGCAGAGAG  33048
LWBQ01000021  415371  ------------------------------------------------------------  415312
GCQL01000547  1037    ------------------------------------------------------------  1096

LWBQ01000158  33049   CATGGGCGATGGGCGCAAACCCGAGTGGGGACACCAGGACTGTGTCATCAGGGCATTGCT  33108
LWBQ01000021  415311  ------------------------------------------------------------  415252
GCQL01000547  1097    ------------------------------------------------------------  1156

LWBQ01000158  33109   TCGGTATGTTTCTACTCCAGCCATGTCGTCTGCGGCTACCCGTCTGCGGCTACCCATTCC  33168
LWBQ01000021  415251  ------------------------------------------------------------  415192
GCQL01000547  1157    TCG                                                           1159

LWBQ01000158  33169   CGCAAAGTACCAAAGTCTAACCAAAGTCCAGATGGTACAAGCGATCATTCTTCACCTGGG  33228
LWBQ01000021  415191  ------------------------------------------------------------  415132
GCQL01000547  1159                                  ------------------------------  1188

LWBQ01000158  33229   TCAACAACCCTCCATGCCCCGTCTGCCTGTCGCCAAGCATCTACCAGGGCATGACGGCGC  33288
LWBQ01000021  415131  ------------------------------------------------------------  415072
GCQL01000547  1189    ------------------------------------------------------------  1248

LWBQ01000158  33289   CGACGCCGGAAGAGAGCGCGCGCGGAGCGCTTCGCGTCGAGCTCTACCGCTGCTCTGCCC  33348
LWBQ01000021  415071  ------------------------------------------------------------  415012
GCQL01000547  1249    ------------------------------------------------------------  1308

LWBQ01000158  33349   AACACTGCGGTGTCTACGAGAGGTTTCCGCGCTACGGCGACGTGTGGTGGCTGCTCCAGA  33408
LWBQ01000021  415011  ------------------------------------------------------------  414952
GCQL01000547  1309    ------------------------------------------------------------  1368

LWBQ01000158  33409   CGCGCCGTGGCCGAGTCGGCGAGTGGGCCAACTGCTTCAGCATGCTGTGCCGCGCCGTCG  33468
LWBQ01000021  414951  ------------------------------------------------------------  414892
GCQL01000547  1369    ------------------------------------------------------------  1428

LWBQ01000158  33469   GCGGAAGGGTGCGGTGGGTGTGGAACGCCGAGGACCACGTGTGGACCGAGGTGTACTCGG  33528
LWBQ01000021  414891  ------------------------------------------------------------  414832
GCQL01000547  1429    ------------------------------------------------------------  1488

LWBQ01000158  33529   AGCACCGCCGACGATGGATCCACGTCGACGCGTGCGAAGAGGCCTGGGACAACCCGCGGC  33588
LWBQ01000021  414831  ------------------------------------------------------------  414772
GCQL01000547  1489    ------------------------------------------------------------  1548

LWBQ01000158  33589   TGTACACGGAAGGTTGGGGCAAGAAGATGTCGTATTGCATCGCATTCTCCATTGACGGGG  33648
LWBQ01000021  414771  ------------------------------------------------------------  414712
GCQL01000547  1549    ------------------------------------------------------------  1608

LWBQ01000158  33649   CCACGGACGTCACCCGGCGATATGTGCGCAAGACCGAGCATGCCAACGACCGCAACCGAT  33708
LWBQ01000021  414711  ------------------------------------------------------------  414652
GCQL01000547  1609    ------------------------------------------------------------  1668

LWBQ01000158  33709   GTCCCGAGGAGGTGCTGCTCCACATTACGCAGGAAATCAAGGGCCTCCGCCGCGCCAACA  33768
LWBQ01000021  414651  ------------------------------------------------------------  414592
GCQL01000547  1669    ------------------------------------------------------------  1728

LWBQ01000158  33769   TGGACAAGGACCAGCGGTTCGCCCTCGAGAAGGAGGACCAGCGGGAGGACCAGGAGCTGC  33828
LWBQ01000021  414591  ------------------------------------------------------------  414532
GCQL01000547  1729    ------------------------------------------------------------  1788

LWBQ01000158  33829   GGGGATACGTGGTGGCATCCATCGCGCAGGCCGTCACGAACCTCGTCCCTGTGGCCTCGG  33888
LWBQ01000021  414531  ------------------------------------------------------------  414472
GCQL01000547  1789    ------------------------------------------------------------  1848

LWBQ01000158  33889   GCTCGGCTCCTACGCGCCGCGCGCCGGCGCCCCCTGGTCACGACGTGAAGCTGCCCGCCG  33948
LWBQ01000021  414471  ------------------------------------------------------------  414412
GCQL01000547  1849    ------------------------------------------------------------  1928

LWBQ01000158  33949   AACGACCCGGGCGGCAAACAGGGTCCGAGGAGTGGATGCTGGCACAGCAGCAAGCTCGGA  34008
LWBQ01000021  414411  ------------------------------------------------------------  414352
GCQL01000547  1929    ------------------------------------------------------------  1968

LWBQ01000158  34009   ATCGGGAGTTTCAACCACCACGCGACCC                                  34036
LWBQ01000021  414351  ----------------------------                                  414324
GCQL01000547  1969    ----------------------------                                  1996
```

Figure S1. Alignments of the genomic sequence of the authentic gene (32562→34036 within LWBQ01000158) for the PNGase family protein and the repetitive copy (414324←415851 within LWBQ01000021) of the *H. sinensis* strain ZJB12195, and the transcriptomic sequence (610→1996 within GCQL01000547) of the strain L0106 [48,51]. The hyphens indicate identical bases, and the spaces denote unmatched sequence gaps.

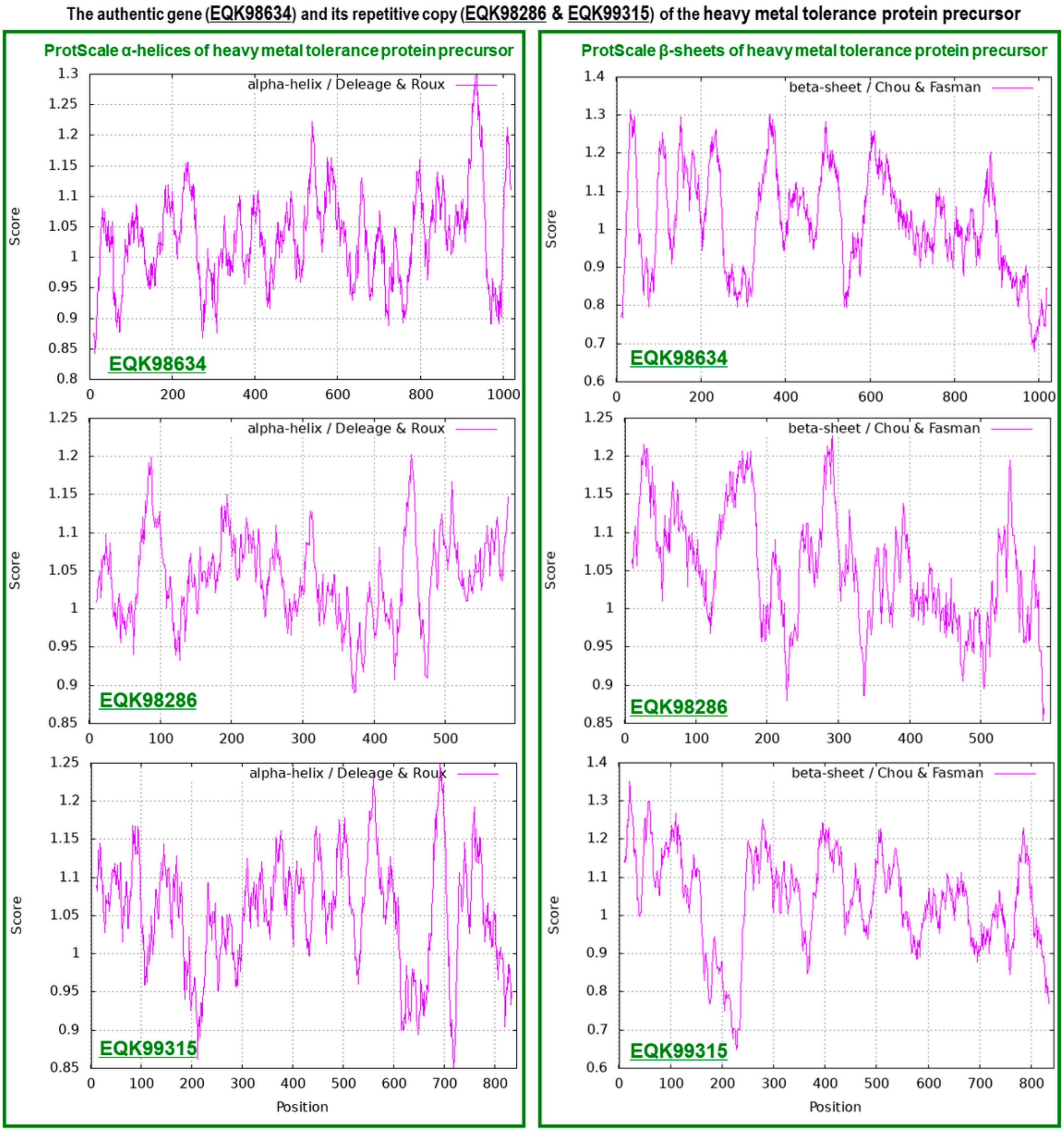

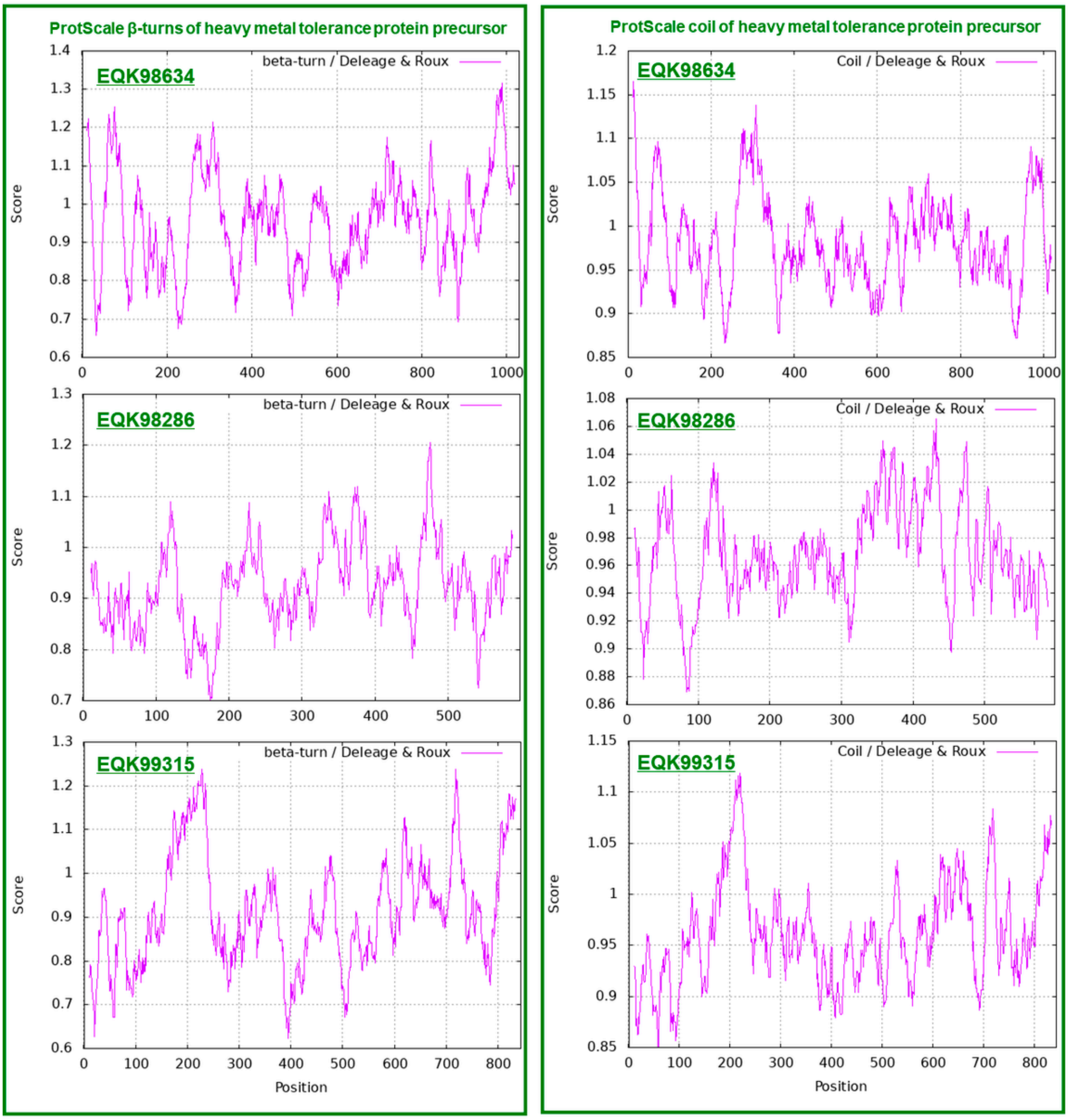

Figure S2. ExPASy ProtScale plots for α-helices (Panel A), β-sheets (Panel B), β-turns (Panel C) and coils (Panel D) of the heavy metal tolerance protein precursor. The protein sequence EQK98634 encoded by the authentic gene of *H. sinensis* strain L0106 was compared with the protein sequence EQK98286 and EQK99315 that encoded by Groups 1 and 2 of the genomic repetitive sequences.

```
EQK98556   101   SKVHRTVIEHARAAASKEQSMTLLQGLKLYPKAVAWSLLISTCIVMEGYDISLVNNFYAF   160
                 S +H + ++ A AA   E++M L + LK+Y KA  WS+ +STCI++EGYD+ ++NN YA+
EQL02995   16    SNMHDSTVQEAAAATENERNMGLFESLKVYRKACMWSVFLSTCIILEGYDLVILNNLYAY   75

EQK98556   161   PQFKRKYGVQLPDGSHEVPAPWQAGLSNGAQVGEIIGLFINGFVSERFGYRYTVLTCLVL   220
                 P F+RK+GV+ PDG+ ++ A WQ+GLSNGA  G+I+GLF NG +++R GYR T++  L
EQL02995   76    PPFQRKFGVEQPDGTFQLTAAWQSGLSNGALCGQILGLFFNGIIADRIGYRKTLIGALTA   135

EQK98556   221   VAAFTAIFSPAANVQTLLVAEILCGIPWGVFQTLAVTYASEVCPIALRSYLTTYVNFCWG   280
                    F  I   A ++  LLV EIL GIPWGVFQTL  TYA+EVCP  LR+YLTTYVN CW
EQL02995   136   CIGFIFILFFAESLPMLLVGEILIGIPWGVFQTLTTTYAAEVCPTHLRAYLTTYVNLCWV   195

EQK98556   281   LGQEIGIGVIRAMLSRDDEWGFRIPYALQWMWPAPLFIGIWFAPESPWWLVRRGRTQDAK   340
                 +GQ +  GV+RAM+ RDD+WG++IP+AL+W+WP PL IGI+ APESPWWLVR GR ++AK
EQL02995   196   IGQFLASGVLRAMILRDDKWGYKIPFALEWIWPIPLIIGIYLAPESPWWLVRNGRLEEAK   255

EQK98556   341   ASLLRLTSLDRETDFDADETVAMMVHTTALEEKMTTGATYLDCFRGVDLRRTELVCMTWA   400
                  SL+RLTS +     F ADET++MMVHT A E+  ++G +Y D F+G +LRRTE+VC+TW
EQL02995   256   QSLIRLTSRNSGVGFRADETLSMMVHTNATEKAASSGTSYTDLFKGTNLRRTEIVCVTWM   315

EQK98556   401   IQNLSGNSFSNYSTYFLKQAGLPEETSYSFALGQYAINMVGVLGAWGLMTLGVGHRSLYL   460
                 +Q L G++F  YSTYF +QAG+  E S++ +L QY +  +G + AW LM++  G R+LYL
EQL02995   316   VQTLCGSTFMGYSTYFYQQAGMAVENSFNMSLAQYGLGAIGTMTAWYLMSVA-GRRTLYL   374

EQK98556   461   YGLCGLCAMLLILGFLGLVPEAHRTEASLATGSVMIVWALIYQLTVGTVCYSLVSELSSR   520
                  G   +C +LL +G        +   A  A GS+++V+   Y  TVG VCYSLVSELSS
EQL02995   375   TGQLTMCGLLLAIGCTSFAGREN-VVAQWAIGSMLLVYTFTYDATVGPVCYSLVSELSST   433

EQK98556   521   RLQIKTVVLGRNLYNTVGIVTGVLTPYMLNPSAWDWSNFAGFFWAGICFLCIIYTYFRLP   580
                 RL+ K+VVL RNLYN VGI T ++TP MLNP+AW+W   +GFFWA  CF+C ++TYFRLP
EQL02995   434   RLRTKSVVLARNLYNIVGITTNIITPRMLNPTAWNWGAKSGFFWACTCFICAVWTYFRLP   493

EQK98556   581   EPRGRTFAELDVLFEKRVSARKFATTDVDVF                                611
                 EP+GR++ ELD+LFE+RVSARKF +T V +F
EQL02995   494   EPKGRSYGELDILFEERVSARKFKSTSVAMF                                524
```

Figure S3. Alignments of the protein sequences EQK98556 and EQL02995 of the *H. sinensis* strain Co18. The protein sequence EQK98556 (636 aa) encoded by the authentic gene for the maltose permease of *H. sinensis* strain Co18 was compared with the protein sequence EQL02995 (550 aa) encoded by the genomic repetitive sequence for a raffinose family of oligosaccharide transporter. The letters and "+" symbols in green in between the sequence lines refer to the identical and conservatively evolved animo acid residues of the protein sequence, respectively, and the spaces indicate non-conservatively variable amino acids of the 2 protein sequences.

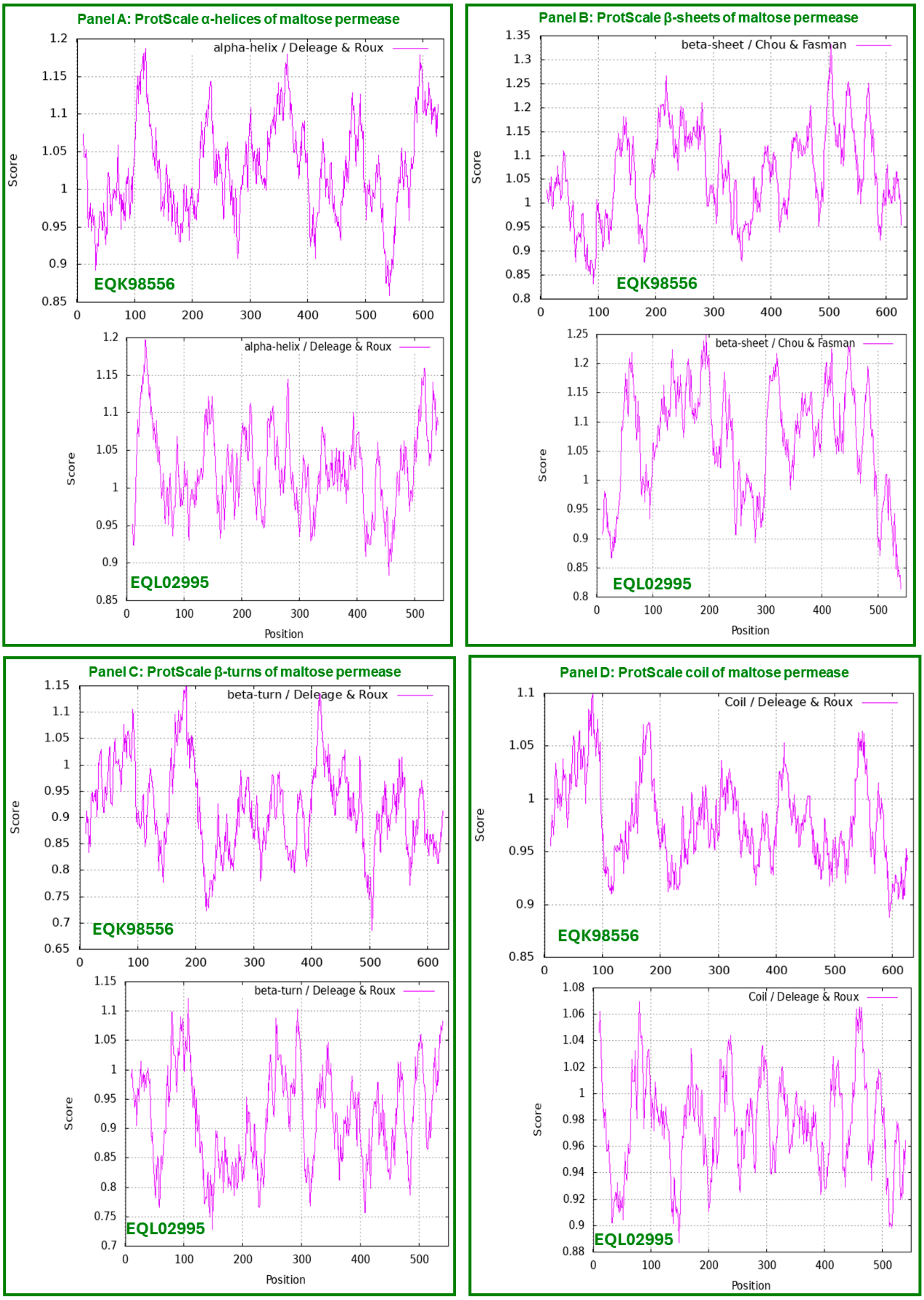
Panel A: ProtScale α-helices of maltose permease
alpha-helix / Deleage & Roux
EQK98556
EQL02995
Score
Position
Panel B: ProtScale β-sheets of maltose permease
beta-sheet / Chou & Fasman
EQK98556
EQL02995
Score
Position
Panel C: ProtScale β-turns of maltose permease
beta-turn / Deleage & Roux
EQK98556
EQL02995
Score
Position
Panel D: ProtScale coil of maltose permease
Coil / Deleage & Roux
EQK98556
EQL02995
Score
Position

Figure S4. ExPASy ProtScale plots for α-helices (Panel A, containing 2 plots in pair), β-sheets (Panel B), β-turns (Panel C) and coils (Panel D) of the triose-phosphate transporter protein. The protein sequence EQK98556 encoded by the authentic gene of *H. sinensis* strain Co18 was compared with the protein sequence EQL02995 encoded by the genomic repetitive sequence.

```
GT#1  AB067721    59        TCGAGTC ACCACTCCC AAA CCCCCTGCGAACACCACAGCAGTTGCCTCGGCGGGACCGCCCCGGCG CCCCAGGGCCCG 136
JAAVMX010000019   19404     ------- --------- --- ----------------------------------------------- ------------ 19481
JAAVMX010000019   32048     ------- --------- --- ----------------------------------------------- ------------ 32125
JAAVMX010000002   18702095  ------- --------- --- -------------------- --------------------------T------------ 18702172
JAAVMX010000018   700       ------- -- ------ --- ----------------------------------T------------ -----------T 776
JAAVMX010000019   6233      ------- ---------C--- ----------------------------------------------- ------------ 6311
JAAVMX010000019   44729     ------- --------- --- ----------------------------------------------- ------------ 44806
GT#2  MG770309
GT#3  HM595984    33        CT----T G-------- --- A----C-T--------------------------------------- ------------ 110
GT#7  AJ488254    464       ------- -A----T-- G--G-------------------------- -------------T-T---- -A-----TG--- 380
GT#8  GU246286    4         ------T --T------ --  ----------------------------------T-------T---- --G----T---- 83
GT#9  GU246288    5         ------TT--------- --- --------------------C----G--------------------- ------------ 84
GT#10 GU246287    6         ------T -G ------ T-- ---------------------T----T-----TTTG----------- -----A------ 82
GT#11 JQ695935    12                  ------- --  ---------- ------------------------------------ ------------ 78
GT#12 GU246296    5         ------T --------- --- ----------------------------------------------- ------------ 83

GT#1  AB067721    137       GACCAGG GCGCCC GCCGGAGGACCCCCAGAC CCTCCTGTCGCA GTGGCATCT CTCAGTCAAGAA GCAAGCAAATGA 213
JAAVMX010000019   19482     ------- ------ ------------------ ------------ --------- ------------ ------------ 19557
JAAVMX010000019   32126     ------- ------ ------------------ ------------ --------- ------------ ------------ 32201
JAAVMX010000002   18702173  ------- ------A------------------ ------------A--------- ---- ------- ------------ 18702249
JAAVMX010000018   777       ------- ------ ------------------ ------------ --------- ---C-------- ------------ 852
JAAVMX010000019   6312      ------- ------ ------T-----------C------------ ---------C------------G------------ 6390
JAAVMX010000019   44807     -------C------ ------------------ ------------ --------- ------------ ------------ 44883
GT#2  MG770309    1                  ----G AT--ATTATT-A----C- -----C-A---- -------T- ------------ ------------ 68
GT#3  HM595984    111       ---T--- ------ ------------------ ------------ -----G--- ------------ ------------ 186
GT#7  AJ488254    379       A------ ------ ------------------ ------------ --------- ------------ ------------ 295
GT#8  GU246286    84        -C-G--- -----T -GAAAT-TT--AA-T--- ------------ ----C---- ------------ ------------ 159
GT#9  GU246288    85        ------- ------ ------------------ ------------ --------- ----------G- -----G------ 160
GT#10 GU246287    83        ------- ------ -----G------------ ----------G  ----GT-T- T----------- AGG--G------ 157
GT#11 JQ695935    79        ------- ------ ------------------ ------------ --------- ------------ ------------ 154
GT#12 GU246296    84        ------- ------ ------------------ ------------ --------- ------------ ------------ 159

GT#1  AB067721    214       ATCAAAACTTTCAACAACGGATCTCTTGGTTCTGGCATC GATG AAGAACGCAGCGAAATGCGATAAGTAATGTGAATTGC 292
JAAVMX010000019   19557     --------------------------------------- ---- ------------------------------------- 19637
JAAVMX010000019   32201     --------------------------------------- ---- ------------------------------------- 32281
JAAVMX010000002   18702249   --------------------------------------C---- ------------------------------------- 18702329
JAAVMX010000018   852       --------------------------------------- ----A------------------------ ------------ 932
JAAVMX010000019   6390      ------------ ---CAC-------------------- ---- --------------- --------- ----------- 6467
JAAVMX010000019   44883     --------------------------------------- ---- ------------------------------------- 44963
GT#2  MG770309    68        --------------------------------------- ---- ----GA---------C -------------------- 147
GT#3  HM595984    186       --------------------------------------- ---- ------------------------------------- 266
GT#7  AJ488254    295       --------------------------------------- ---- -----------A------------------------- 224
GT#8  GU246286    159       -------------T-----------------T------- ---- -G---------------G------------------- 239
GT#9  GU246288    161       ------------------------T-------------- ---- ------------------------------------- 240
GT#10 GU246287    158       ------T-----------------T-------------- ---- ------C----C------------G------------ 237
GT#11 JQ695935    155       --------------------------------------- ---- ------------------------------------- 234
GT#12 GU246296    160       -----------------------------------------------------------------G---------------- 239

GT#1  AB067721    293       AGAATTCAGTGAACCA TCGAATCTTT GAACG CACATT G CGCCCGCCAGCACTCTGGCGGGCATGC  CTGTCCGAGC 367
JAAVMX010000019   19638     ---------------- ---------- ----- ------ - ---------------------------  ---------- 19712
JAAVMX010000019   32282     ---------------- ---------- ----- ------ - ---------------------------  ---------- 32356
JAAVMX010000002   18702330  ---------------- ----------T----- -----  - ------- -------------------  ---------- 18702403
JAAVMX010000018   933       ---------------  C---------- -----C------ -C------------------- -------  ---------- 1005
JAAVMX010000019   6468      ----------------C---------- ----- ------ -C---------------------------  ---------- 6544
JAAVMX010000019   44964     ---------------- ---------- ----- ------ - ---------------------------CT---------- 45040
GT#2  MG770309    148       ---------------- ---------- ----- ------ - ---------------------------  ---------- 222
GT#3  HM595984    267       ---------------- ---------- ----- ------ - ----------------A----------  ---------- 341
GT#7  AJ488254    223       ---------------- ---------- ----- A----- - ---------------------------  ---------- 147
GT#8  GU246286    240       ---------------- --T-T-T--- ----- ------ - ----------------------A----  ---------- 314
GT#9  GU246288    241       ---------------- ---------- ----- ------ - -----------C---------------  ---------- 315
GT#10 GU246287    238       ---------------- ---------- ----- ------G- G---G----------------------  T--------- 313
GT#11 JQ695935    235       ---------------- ---------- ----- ------ - ---------------------------  ---------- 309
GT#12 GU246296    240       ---------------- ---------- ----- ------ - ---------------------------  ---------- 314

GT#1  AB067721    368       GTCATCTCAACCCTCGAG CCCCCCGCCTC GCGG  C GGCGGGG CCCGGCCTT GGGGGTCACGGCC CCGC  GCCG 438
JAAVMX010000019   19713     ------------------  ---------- ----  - ------- --------- ------------- ----  ---- 19782
JAAVMX010000019   32357     ------------------  ---------- ----  - ------- --------- ------------- ----  ---- 32426
JAAVMX010000002   18702404  ------------------ ----- ----- ----  - ------- --------- ------------- ----  ---- 18702473
JAAVMX010000018   1006      ------------------ ------ ---- ----  - ------- ---------G------------- ----   --- 1075
JAAVMX010000019   6545      ------------------  ---------- ----  - ------- ---- ---- -------------C----  ---- 6614
JAAVMX010000019   45041     ------------------C----------A ----TA- -CG---C --------- ------TC-----AA---  ----T 45123
GT#2  MG770309    223       ------------------ ----------- ----  - ------- --------- ------------- ----  ---- 293
GT#3  HM595984    342       ------------------  ---------- ----  - -A----- --------- ------------- -T--  ---- 412
GT#7  AJ488254    148       ------------------ ----A-A----T---A  -T------- --------- ------------- ----  ---- 76
GT#8  GU246286    315       ------------------ ----------- ----  - -A----- --------- -------G----- ----  C--C 387
GT#9  GU246288    316       ------------------ ----------- -G--  - ------- --------- ------------- ----  ---- 388
GT#10 GU246287    314       ----A--A----A----- ----------T -G--  T ------- ------T-- T-----T--C--- --C-  C--C 384
GT#11 JQ695935    310       ------------------ ----------- ----  - ------- --TTCT--- --TT---CTTTAT TGAT  -A-- 381
GT#12 GU246296    315       ------------------ ----------- ----  - -------C--------- ------------- --C-CGC--- 388

GT#1  AB067721    439         CCCCC  T AAACGC AG       TGGCGACC  CC  GCCGCGGCTCC CC TGCGCAGTAGCT  CG  CTGAGAAC 497
JAAVMX010000019   19783       -----  - ------ --       --------  --  ----------- -- ------------  --  -------- 19842
JAAVMX010000019   32427       -----  - ------ --       --------  --  ----------- -- ------------  --  -------- 32486
JAAVMX010000002   18702474    -----  - ------ --       --------  --  ----------- -- ------------  --  -------- 18702532
JAAVMX010000018   1076        -----  - ------ --       --------  --  ----------- -- ------------  --  -------- 1134
JAAVMX010000019   6614        -----  - ------G--G      --------GC--  ----------- -- ------------  --GCT------- 6678
JAAVMX010000019   45125     CCT--- G - ----- --CCTAAGTG-------   --  --GA-------A-- --T-A-------CA--  -------- 45195
GT#2  MG770309    294         -----  - --                                                                      301
GT#3  HM595984    413         -----  - ---T-- --       --------  --  ----------- -- ------------  --  -C---G-- 479
GT#7  AJ488254    75          -----  - ------ --       --------  --  ----------- -T ------C-----  G-  -------- 18
GT#8  GU246286    388         ----T  - ------ --       G-------  --  ----- ---T- A- ------------  -A  -C------ 443
GT#9  GU246288    389         -----  - ------ --       G-------  --  ----------- -- --- -C-G-A--  -A  -C------ 452
GT#10 GU246287    385         -----CC-A------ G-       G--G-C--  --  C--C--C-C-- --T--GC-----TT-  AA  -C-G---- 417
GT#11 JQ695935    382        G-TTG   - -CTT-G --      G-C-T- --GG-T   -TCG- ----GG- - -TC-GA--  T-  -ATTAGTG 445
GT#12 GU246296    361         -----  -A------ G-       G-------  --CG--G-------- -- -----G------  -AAC-GA----- 420

GT#1  AB067721    498       CT CGCACCGG  GA GCGCGGA     GGCGG TCACGCC GTG     AA ACCACC   ACACCC TCCA          549
JAAVMX010000019   19843     -- --------  -- -------     ----- ----- -  --G    -- ------   ------ ----          19894
JAAVMX010000019   32487     -- --------  -- -------     ----- ----- -  --G    -- ------   ------ ----          32537
JAAVMX010000002   18702533  -- --------  -- -------     ----- ------- ---TG   -- ------   ------ ----          18702586
JAAVMX010000018   1135      -- --------  -- -------     ----- ----- -  --G    -- ------   ------ ----          1186
JAAVMX010000019   6679      --C--------  -- -------     ----- ----- -  --G    -- ------ACA------ ----          6733
JAAVMX010000019   45196     -- --------  -- -------CCCAT-----C-------  --GGATG--C------ACC------ ----          45251
GT#2  MG770309    301
GT#3  HM595984    480       -C --------  A- ------G     --T-- -------   -C-   -- ------   ---     ---          519
GT#7  AJ488254    17        --T-----                                                                        9
GT#8  GU246286    444       T- -------A  A- ---- --     A---- -T----- ---     -- -A-T--   --G--T ----          494
GT#9  GU246288    453       -- --------  --G-------     ----- ------- -G-     -- - ----   --G--- - --          496
GT#10 GU246287    418       -C ---C----AA-- -G-G---     --G--GG- --                                         478
GT#11 JQ695935    446       -- AA-GT     T- C-- -CC     --TTCGC-GT-AT AAT    T-TCTG  --   TTG-   -G-G          485
GT#12 GU246296    421       -- --------  --G-G-----     ---G---------G---    A-- ------   --G---T----          480
```

Figure S5. Alignment of repetitive ITS copies within the genome JAAVMX000000000 of the *H. sinensis* strain IOZ07 and ITS sequences of GC-biased genotypes of *O. sinensis*. The genome segments JAAVMX010000002, JAAVMX010000018, and JAAVMX010000019 were obtained from the *H. sinensis* strain IOZ07 [49]. One copy each within JAAVMX010000002 and JAAVMX010000018, indicated in **green**, shares 97.4% or 97.0% similarity with AB067721. JAAVMX010000019 contains 4 repetitive ITS copies, including 2 sequences in **black** (19404→19894 and 32048→32537), which are 100% identical to AB067721, and 2 other sequences in **blue** (6233→6733 and 44729→45251), with 94.5% and 90.8% similarity to AB067721. "GT" denotes the *O. sinensis* genotype. AB067721, MG770309, HM595984, AJ488254, GU246286, GU246288, GU246287, JQ695935, and GU246296 are the ITS sequences in pink of GC-biased Genotypes #1–3 and #7–12 of *O. sinensis*, respectively. The underlined sequence in **black** represents the 5.8S gene of GC-biased Genotype #1 *H. sinensis*. The hyphens indicate identical bases, and the spaces denote unmatched sequence gaps.

References

[1] Zhu J-S, Halpern GM, Jones K. The scientific rediscovery of an ancient Chinese herbal medicine: *Cordyceps sinensis*: Part I. J. Altern. Complem. Med. 1998a; 4(3): 289–303. DOI: 10.1089/acm.1998.4.3-289

[2] Zhu J-S, Halpern GM, Jones K. The scientific rediscovery of a precious ancient Chinese herbal regimen: *Cordyceps sinensis*: Part II. J. Altern. Complem. Med. 1998b; 4(4): 429–457. DOI: 10.1089/acm.1998.4.429

[3] Zhu J-S, Li C-L, Tan N-Z, Berger JL, Prolla TA. Combined use of whole-gene expression profiling technology and mouse lifespan test in anti-aging herbal product study. Proc. 2011 New TCM Products Innovation and Industrial Development Summit, Hangzhou, China. Nov 27, 2011. pp. 443–448. https://xueshu.baidu.com/usercenter/paper/show?paperid=08341c17fa58c8f85584b92572b90f75&site=xueshu_se

[4] Li X-Z, Li Y-L, Yao Y-S, Xie W-D, Zhu J-S. Molecular identification of *Ophiocordyceps sinensis* genotypes and the indiscriminate use of the Latin name for the multiple genotypes and the natural insect-fungi complex. Am. J. Biomed. Sci. 2022a; 14(3): 115–135. doi: 10.5099/aj220300115

[5] Li Y-L, Li X-Z, Yao Y-S, Wu Z-M, Gao L, Tan N-Z, Luo Z-Q, Xie W-D, Wu J-Y, Zhu J-S. Differential cooccurrence of multiple genotypes of *Ophiocordyceps sinensis* in the stromata, stromal fertile portion (ascocarps) and ascospores of natural *Cordyceps sinensis*. PLoS ONE. 2023b: 18(3): e0270776. https://doi.org/10.1371/journal.pone.0270776

[6] Li C-L. A study of *Tolypocladium sinense* C.L. Li. Sp. nov and cylosporum production. Acta Mycolog. Sinica. 1988; 7(2): 93–98.

[7] Dai R-Q, Lan J-L, Chen W-H, Li X-M, Chen Q-T, Shen C-Y. Discovery of a new fungus *Paecilomyces hepiali* Chen & Dai. Acta Agric. Univ. Pekin. 1989; 15, 221–224.

[8] Jiang Y, Yao Y-J. Anamorphic fungi related to *Cordyceps sinensis*. Mycosystema. 2003; 22(1): 161−176.

[9] Chen Y-Q, Hu B, Xu F, Zhang W, Zhou H, Qu L-H. Genetic variation of *Cordyceps sinensis*, a fruit-body-producing entomopathogenic species from different geographical regions in China. FEMS Microbiol, Lett. 2004; 230: 153–158. Doi: 10.1016/S0378-1097(03)00889-9

[10] Zhang Y-J, Sun B-D, Zhang S, Wang M, Liu X-Z, Gong W-F. Mycobiotal investigation of natural *Ophiocordyceps sinensis* based on culture-dependent investigation. Mycosystema. 2010; 29: 518–527.

[11] Zhang S-W, Cem K, Liu Y, Zhou X-W, Wang C-S. Metatranscriptomics analysis of the fruiting caterpillar fungus collected from the Qinghai-Tibetan plateau. Sci. Sinica Vitae. 2018; 48(5): 562. doi: 10.1360/N052017-00253

[12] Barseghyan GS, Holliday JC, Price TC, Madison LM, Wasser SP. Growth and cultural-morphological characteristics of vegetative mycelia of medicinal caterpillar fungus *Ophiocordyceps sinensis* G.H. Sung *et al*. (Ascomycetes) isolates from Tibetan Plateau (P.R.China). Intl. J. Med. Mushrooms. 2011; 13(6): 565−581. doi: 10.1615/intjmedmushr.v13.i6.90

[13] Xia F, Liu Y, Shen G-R, Guo L-X, Zhou X-W. Investigation and analysis of microbiological communities in natural *Ophiocordyceps sinensis*. Can. J. Microbiol. 2015; 61: 104−111. http://dx.doi.org/10.1139/cjm-2014-0610

[14] Wang Y, Liu Y-F, Tang D-X, Wang Y-B, Adams AEM, Yu H. *Tolypocladium reniformisporum* sp. nov. and *Tolypocladium cylindrosporum* (Ophiocordycipitaceae, Hypocreales) co-occurring on *Ophiocordyceps sinensis*. Mycol. Progress. 2022; 21: 199–214. https://doi.org/10.1007/s11557-021-01675-y

[15] Engh IB. Molecular phylogeny of the *Cordyceps-Tolypocladium* complex. Candidate scientific thesis, Department of Biology, University of Oslo, Oslo, Norway, 1999.
[16] Kinjo N, Zang M. Morphological and phylogenetic studies on *Cordyceps sinensis* distributed in southwestern China. Mycosci. 2001; 42: 567–574.
[17] Liu Z-Y, Yao Y-J, Liang Z-Q, Liu A-Y, Pegler DN, Chase MW. Molecular evidence for the anamorph-teleomorph connection in *Cordyceps sinensis*. Mycol. Res. 2001; 105: 827–832. https://doi.org/10.1017/S095375620100377X
[18] Stensrud Ø, Hywel-Jones NL, Schumacher T. Towards a phylogenetic classification of Cordyceps: ITS nrDNA sequence data confirm divergent lineages and paraphyly. Mycol. Res. 2005; 109(1): 41–56. DOI: 10.1017/S095375620400139X
[19] Stensrud Ø, Schumacher T, Shalchian-Tabrizi K, Svegården IB, Kauserud H. Accelerated nrDNA evolution and profound AT bias in the medicinal fungus *Cordyceps sinensis*. Mycol. Res. 2007; 111: 409–415. https://doi.org/10.1016/j.mycres.2007.01.015
[20] Wei X-L, Yin X-C, Guo Y-L, Shen N-Y, Wei J-C. Analyses of molecular systematics on *Cordyceps sinensis* and its related taxa. Mycosystema. 2006; 25(2): 192–202. https://europepmc.org/article/cba/618538
[21] Wei J-C, Wei X-L, Zheng W, Guo W, Liu R. Species identification and component detection of *Ophiocordyceps sinensis* cultivated by modern industry. Mycosystema. 2016; 35: 404–410.
[22] Xiao W, Yang J-L, Zhu P, Cheng K, He H, Zhu H, Wang Q. Non-support of species complex hypothesis of *Cordyceps sinensis* by targeted rDNA-ITS sequence analysis. Mycosystema. 2009; 28: 724–730.
[23] Zhang Y-J, Xu L, Zhang S, Liu X-Z, An Z, WangMu, Guo Y-L. Genetic diversity of *Ophiocordyceps sinensis*, a medicinal fungus endemic to the Tibetan Plateau: implications for its evolution and conservation. BMC Evolut. Biol. 2009; 9: 290. http://www.biomedcentral.com/1471-2148/9/290
[24] Zhu J-S, Guo Y-L, Yao Y-S, Zhou Y-J, Lu J-H, Qi Y, Liu X-J, Wu Z-M, Chen W, Zhang L, Yin W-T, Zheng T-Y, Zhang L-J. Maturation of *Cordyceps sinensis* associates with co-existence of *Hirsutella sinensis* and *Paecilomyces hepiali* DNA and dynamic changes in fungal competitive proliferation predominance and chemical profiles. J. Fungal Res. 2007; 5(4): 214–224. DOI: 10.3969/j.issn.1672-3538.2007.04.009
[25] Chen C-S, Hseu R-S, Huang C-T. Quality control of *Cordyceps sinensis* teleomorph, anamorph, and Its products. Chapter 12, *in Quality Control of Herbal Medicines and Related Areas*; Shoyama, Y., Ed.; InTech: Rijeka, Croatia. 2011; pp. 223–238. www.intechopen.com
[26] Gao L, Li X-H, Zhao J-Q, Lu J-H, Zhao J-G, Zhu J-S. Maturation of *Cordyceps sinensis* associates with alterations of fungal expressions of multiple *Ophiocordyceps sinensis* mutants with transition and transversion point mutations in stroma of *Cordyceps sinensis*. Beijing Da Xue Xue Bao. 2012; 44(3): 454–463. doi: 10.3969/j.issn.1671-167X.2012.03.025
[27] Mao X-M, Zhao S-M, Cao L, Yan X, Han R-X. The morphology observation of *Ophiocordyceps sinensis* from different origins. J. Environ. Entomol. 2013; 35: 343–353. doi: 10.3969/j.issn.1674-0858.2013.03.11
[28] Li Y-L, Gao L, Yao Y-S, Li X-Z, Wu Z-M, Tan N-Z, Luo Z-Q, Xie W-D, Wu J-Y, Zhu J-S. Altered GC- and AT-biased genotypes of *Ophiocordyceps sinensis* in the stromal fertile portions and ascospores of natural *Cordyceps sinensis*. PLoS ONE. 2023a; 18(6): e0286865. https://doi.org/10.1371/journal.pone.0286865
[29] Yao Y-S, Zhu J-S. Indiscriminate use of the Latin name for natural *Cordyceps sinensis* and *Ophiocordyceps sinensis* fungi. Chin. J. Chin. Mater. Med. 2016; 41(7): 1316–1366.
[30] Li X-Z, Li Y-L, Yao Y-S, Xie W-D, Zhu J-S. Further discussion with Li *et al*. (2013, 2019) regarding the "ITS pseudogene hypothesis" for *Ophiocordyceps sinensis*. Mol. Phylogenet. Evol. 2020a; 146: 106728. DOI:10.1016/j.ympev.2019.106728
[31] Sung G-H, Hywel-Jones NL, Sung J-M, Luangsa-ard JJ, Shrestha B, Spatafora JW. Phylogenetic classification of *Cordyceps* and the clavicipitaceous fungi. Stud. Mycol. 2007; 57: 5–59.
[32] Hawksworth DL, Crous PW, Redhead SA, Reynolds DR, Samson RA, Seifert KA, and 82 other authors. The Amsterdam declaration on fungal nomenclature. IMA Fungus. 2011; 2: 105−112. DOI: 10.5598/imafungus.2011.02.01.14
[33] Taylor JW. One Fungus = One Name: DNA and fungal nomenclature twenty years after PCR. IMA Fungus. 2011; 2(2): 113–120.
[34] Zhang S, Zhang Y-J, Shrestha B, Xu J-P, Wang C-S, Liu X-Z. *Ophiocordyceps sinensis* and *Cordyceps militaris*: research advances, issues, and perspectives. Mycosystema. 2013b; 32: 577−597. DOI: 10.13346/j.mycosystema.2013.04.001
[35] Zhang Y-J, Li E-W, Wang C-S, Li Y-L, Liu X-Z. *Ophiocordyceps sinensis*, the flagship fungus of China: terminology, life strategy and ecology. Mycol. 2012; 3: 2–10. DOI: 10.1080/21501203.2011.654354
[36] Zhang Y-J, Zhang S, Li Y-L, Ma S-L, Wang C-S, Xiang M-C, Liu X, An Z-Q, Xu J-P, Liu X-Z. Phylogeography and evolution of a fungal–insect association on the Tibetan Plateau. Mol. Ecol. 2014; 23: 5337–5355. doi: 10.1111/mec.12940

[37] Zhu J-S, Gao L, Li X-H, Yao Y-S, Zhao J-Q. Maturational alteration of oppositely orientated rDNA and differential proliferation of GC- and AT-biased genotypes of *Ophiocordyceps sinensis* and *Paecilomyces hepiali* in natural *Cordyceps sinensis*. Am. J. Biomed. Sci. 2010; 2: 217–238. doi: 10.5099/aj100300217

[38] Zhu J-S, Li Y-L, Yao Y-S, Xie W-D. The multiple genotypes of *Ophiocordyceps sinensis* and the ITS pseudogene hypothesis. Mol. Phylogenet. Evol. 2019; 139: 106322. https://doi.org/10.1016/j.ympev.2018.10.034

[39] Zhang S, Zhang Y-J, Liu X-Z, Zhang H, Liu D-S. On the reliability of DNA sequences of *Ophiocordyceps sinensis* in public databases. J. Ind. Microbiol. Biotechnol. 2013a; 40: 365–378. DOI 10.1007/s10295-012-1228-4

[40] Bushley KE, Li Y, Wang W-J, Wang X-L, Jiao L, Spatafora JW, Yao Y-J. Isolation of the MAT1-1 mating type idiomorph and evidence for selfing in the Chinese medicinal fungus *Ophiocordyceps sinensis*. Fungal Biol. 2013; 117: 599–610. http://dx.doi.org/10.1016/j.funbio.2013.06.001

[41] Li Y, Jiao L, Yao Y-J. Non-concerted ITS evolution in fungi, as revealed from the important medicinal fungus *Ophiocordyceps sinensis*. Mol. Phylogenetics Evol. 2013; 68: 373–379. http://dx.doi.org/10.1016/j.ympev.2013.04.010

[42] Li Y, Jiang L, Wang K, Wu H-J, Yang R-H, Yan Y-J, Bushley KE, Hawksworth DL, Wu Z-J, Yao Y-J. RIP mutated ITS genes in populations of *Ophiocordyceps sinensis* and their implications for molecular systematics. IMA Fungus. 2020b; 11: 18. DOI:10.1016/j.mimet.2016.06.025

[43] Li X-Z, Xiao M-J, Li Y-L, Gao L, Zhu J-S. Mutations and differential transcription of mating-type and pheromone receptor genes in *Hirsutella sinensis* and the natural *Cordyceps sinensis* insect–fungi complex. Biol (Basel). 2024; 13(8): 632. https://doi.org/10.3390/biology13080632

[44] Hu X, Zhang Y-J, Xiao G-H, Zheng P, Xia Y-L, Zhang X-Y, St Leger RJ, Liu X-Z, Wang C-S. Genome survey uncovers the secrets of sex and lifestyle in caterpillar fungus. Chin. Sci. Bull. 2013; 58: 2846–2854. doi: 10.1007/s11434-013-5929-5

[45] Zhang S, Zhang Y-J. Molecular evolution of three protein-coding genes in the Chinese caterpillar fungus *Ophiocordyceps sinensis*. Microbiol. Chin. 2015; 42(8): 1549–1560. DOI: 10.13344/j.microbiol.china.150279

[46] Li Y, Hsiang T, Yang R-H, Hu X-D, Wang K, Wang W-J, Wang X-L, Jiao L, Yao Y-J. Comparison of different sequencing and assembly strategies for a repeat-rich fungal genome, *Ophiocordyceps sinensis*. J. Microbiol. Methods. 2016a; 128: 1–6. http://dx.doi.org/10.1016/j.mimet.2016.06.025

[47] Jin L-Q, Xu Z-W, Zhang B, Yi M, Weng C-Y, Lin S, Wu H, Qin X-T, Xu F, Teng Y, Yuan S-J, Liu Z-Q, Zheng Y-G. Genome sequencing and analysis of fungus *Hirsutella sinensis* isolated from *Ophiocordyceps sinensis*. AMB Expr. 2020; 10: 105. DOI: 10.1186/S13568-020-01039-x

[48] Liu J, Guo L-N, Li Z-W, Zhou Z, Li Z, Li Q, Bo X-C, Wang S-Q, Wang J-L, Ma S-C, Zheng J, Yang Y. Genomic analyses reveal evolutionary and geologic context for the plateau fungus *Ophiocordyceps sinensis*. Clin. Med. 2020; 15: 107–119. DOI: 10.1186/s13020-020-00365-3

[49] Shu R-H, Zhang J-H, Meng Q, Zhang H, Zhou G-L, Li M-M, Wu P-P, Zhao Y-N, Chen C, Qin Q-L. A new high-quality draft genome assembly of the Chinese cordyceps *Ophiocordyceps sinensis*. Genome Biol. Evol. 2020; 12(7): 1074–1079. DOI: 10.1093/gbe/evaa112

[50] Will I, Das B, Trinh T, Brachmann A, Ohm RA, de Bekker C. Genetic underpinnings of host manipulation by *Ophiocordyceps* as revealed by comparative transcriptomics. G3 Genes|Genomes|Genetics (Bethesda). 2020; 10: 2275–2296. doi: 10.1534/g3.120.401290

[51] Liu Z-Q, Lin S, Baker PJ, Wu L-F, Wang X-R, Wu H, Xu F, Wang H-Y, Brathwaite ME, Zheng Y-G. Transcriptome sequencing and analysis of the entomopathogenic fungus *Hirsutella sinensis* isolated from *Ophiocordyceps sinensis*. BMC Genome. 2015;16: 106. DOI 10.1186/s12864-015-1269-y

[52] Yang J-Y, Anishchenko I, Park H, Peng Z-L, Ovchinnikov S, Baker D, Improved protein structure prediction using predicted interresidue orientations. PNAS, 2020; 117(3): 1496–1503. https://doi.org/10.1073/pnas.1914677117

[53] Mallick SB, Das S, Venkatasubramanian A, Kundu S, Datta PP. Comprehensive *in silico* analyses of fifty-one uncharacterized proteins from *Vibrio cholerae*. PLoS ONE. 2024; 19(10): e0311301. https://doi.org/10.1371/journal.pone.0311301

[54] Chou P-Y, Fasman G-D. Prediction of the secondary structure of proteins from their amino acid sequence. Adv. Enzymol. Relat. Areas. Mol. Biol. 1978; 47: 45–148. DOI: 10.1002/9780470122921.ch2

[55] Deleage G, Roux B. An algorithm for protein secondary structure prediction based on class prediction. Protein Engineering, Design and Selection 1987; 1: 289–294. https://doi.org/10.1093/protein/1.4.289

[56] Gasteiger E, Hoogland C, Gattiker A, Duvaud S, Wilkins MR, Appel RD, Bairoch A. Protein Identification and Analysis Tools on the Expasy Server. Chapter 52 (*In*) John M. Walker (*ed*): The Proteomics Protocols Handbook, Humana Press (2005). pp. 571–607.

[57] Chahinian H, Sarda L. Distinction between esterases and lipases: comparative biochemical properties of sequence-related carboxylesterases. Protein & Peptide Lett. 2009; 16(10): 1149-1161. doi: 10.2174/092986609789071333

[58] Li Y, Wang Y-H, Wang K, Yang R-H, Jiao L, Yao Y-J. Response to "The multiple genotypes of *Ophiocordyceps*

sinensis and the ITS pseudogene hypothesis". Mol. Phylogenetics Evol. 2019; 139: 106522. https://doi.org/10.1016/j.ympev.2019.106522
[59] Xiao Y-Y, Chen C, Dong J-F, Li C-R, Fan M-Z. Morphological observation of ascospores of *Ophiocordyceps sinensis* and its anamorph in growth process. J. Anhui Agricult. Univ. 2011; 38(4): 587–591. DOI: 10.13610/j.cnki.1672-352x.2011.04.022
[60] Xiang L, Li Y, Zhu Y-J, Luo H-M, Li C-F, Xu X-L, Sun C, Song J-Y, Shi L-C, He L, Sun W, Chen S-L. Transcriptome analysis of the *Ophiocordyceps sinensis* fruiting body reveals putative genes involved in fruiting body development and cordycepin biosynthesis. Genomics. 2014; 103: 154–159. http://dx.doi.org/10.1016/j.ygeno.2014.01.002
[61] Xia E-H, Yang D-R, Jiang J-J, Zhang Q-J, Liu Y, Liu Y-L, Zhang Y, Zhang H-B, Shi C, Tong, Y, Kim C, Chen H, Peng Y-Q, Yu Y, Zhang W, Eichler EE, Gao L-Z. The caterpillar fungus, *Ophiocordyceps sinensis*, genome provides insights into highland adaptation of fungal pathogenicity. Sci. Rep. 2017; 7: 1806. doi: 10.1038/s41598-017-01869-z
[62] Selker EU. Epigenetic phenomena in filamentous fungi: useful paradigms or repeat-induced confusion? Trends Genet. 1997; 13: 296–301. DOI: 10.1016/S0168-9525(97)01201-8
[63] Selker EU, Stevens JN. DNA methylation at asymmetric sites is associated with numerous transition mutations. Proc. Natl. Acad. Sci. USA. 1985; 82: 8114–8118. DOI: 10.1073/pnas.82.23.8114
[64] Selker EU. Premeiotic instability of repeated sequences in Neurospora crassa. Annu. Rev. Genet. 1990; 24: 579–613. DOI: 10.1146/annurev.ge.24.120190.003051
[65] Selker EU. Repeat-induced gene silencing in fungi. Adv. Genet. 2002; 46: 439–450. DOI: 10.1016/S0065-2660(02)46016-6
[66] Gladyshev E, Kleckner N. DNA sequence homology induces cytosine-to-thymine mutation by a heterochromatin-related pathway in Neurospora. Nat Genet. 2017; 49(6): 887–894. doi: 10.1038/ng.3857
[67] Hane JK, Williams AH, Taranto AP, Solomon PS, Oliver RP. Repeat-induced point mutation: A fungal-specific, endogenous mutagenesis process. In: van den Berg M, Maruthachalam K (eds) Genetic transformation systems in fungi, Volume 2. Fungal Biology. Springer, Cham. 2015. https://doi.org/10.1007/978-3-319-10503-1_4
[68] Hood ME, Katawczik M, Giraud T. Repeat-induced point mutation and the population structure of transposable elements in *Microbotryum violaceum*. Genetics. 2005; 170(3): 1081-1089. doi: 10.1534/genetics.105.042564
[69] Sun X-G. Genome wide spectrum of somatic mutation and RIP in *Neurospora crassa*. Ph.D. candidate thesis, School Life Sci, Nanjing Univ, Nanjing, China, 2021.
[70] Sun H-Z, Ge S. Review of the Evolution of Duplicated Genes. Chin Bull Bot. 2010; 45: 13–22. doi: 10.3969/j.issn.1674-3466.2010.01.002
[71] Yang Z-H, Bai C-J, Pu Y-W, Kong Q-H, Guo Y-B, Ouzhuluobud, Gengdeng, Liu X-Y, Zhao Q, Qiu Z-C, Zheng W-S, He Y-X, Lin Y-H, Deng L, Zhang C, Xu S-H, Peng Y, Xiang K, Zhang X-M, Baimayangji, Cirenyangji, Cui C, Baimakangzhuo, Gonggalanzi, Bianba, Pan Y-Y, Xin J-X, Wang Y, Liu S-M, Wang L-B, Guo H-L, Feng Z-Z, Wang S-B, Shi H, Jiang B-H, Wu T-Y, Qi X-B, Su B. Genetic adaptation of skin pigmentation in highland Tibetans. PNAS (Anthropol). 2022; 119(40): e2200421119. https://doi.org/10.1073/pnas.2200421119
[72] Li Y-L, Yao Y-S, Zhang Z-H, Xu H-F, Liu X, Ma S-L, Wu Z-M, Zhu J-S. Synergy of fungal complexes isolated from the intestines of *Hepialus lagii* larvae in increasing infection potency. J. Fungal Res. 2016b; 14: 96–112.
[73] Dong R, Chen L-L, Yang L. Circular RNA studies in the post-genomic era. Chin, J, Cell Biol. 2014a; 36(11): 1455–1459. DOI: 10.11844/cjcb.2014.11.9001
[74] Dong Y-Z, Zhang L-J, Wu Z-M, Gao L, Yao Y-S, Tan N-Z, Wu J-Y, Ni L-Q, Zhu J-S. Altered proteomic polymorphism in the caterpillar body and stroma of natural *Cordyceps sinensis* during maturation. PLoS ONE. 2014b; 9(10): e109083. doi:10.1371/journal.pone.0109083
[75] Huelsenbeck JP, Ronquist F. MRBAYES: Bayesian inference of phylogeny. Bioinformat. 2001; 17: 754–755. DOI: 10.1093/bioinformatics/17.8.754
